# *Lactobacillus* GG (LGG) and smectite versus LGG alone for acute gastroenteritis: a double-blind, randomized controlled trial

**DOI:** 10.1007/s00431-012-1878-2

**Published:** 2012-11-01

**Authors:** Małgorzata Pieścik-Lech, Magdalena Urbańska, Hania Szajewska

**Affiliations:** Department of Paediatrics, The Medical University of Warsaw, Dzialdowska 1, 01-183 Warsaw, Poland

**Keywords:** LGG, Diosmectite, Dioctahedral smectite, Diarrhea, Infants, RCT

## Abstract

Diarrhea treatment with either *Lactobacillus* GG (LGG) or smectite as an adjuvant to standard rehydration therapy has proven efficacy. In countries where both LGG and smectite are available, concomitant use is frequently practiced. We investigated whether LGG plus smectite is superior to LGG alone in the management of children with acute gastroenteritis (AGE). A double-blind, placebo-controlled, randomized trial was performed. Children aged 4 to 60 months with AGE received LGG 6 × 10^9^ colony forming units/day plus randomly either smectite (3 g) or placebo as an adjuvant to the standard rehydration therapy. Of the 88 children randomized, 81 (92 %) were available for intention-to-treat analysis. The duration of diarrhea in the LGG/smectite group (*n* = 44) compared with the LGG/placebo group (*n* = 37) was similar (*P* = 0.43). There were no significant differences between the study groups for the secondary outcomes, with three exceptions. On day 4, in the LGG/placebo group compared to the LGG/smectite group, there was significantly reduced stool frequency (*P* = 0.03). While there was a significant (*P* = 0.05) difference in stool consistency on the Bristol Stool Form Scale on day 4, it was not of clinical relevance. Finally, in the LGG/smectite group compared to the LGG/placebo group, there was a significantly shorter duration of intravenous therapy after randomization (*P* = 0.02). No adverse events were observed in the study groups. *Conclusion*: LGG plus smectite and LGG alone are equally effective for treating young children with AGE. Combined use of the two interventions is not justified.

## Introduction

It is generally recommended that oral rehydration should be used as first-line therapy to prevent or treat dehydration in children with acute gastroenteritis (AGE) [5]. Despite the proven efficacy of oral rehydration therapy, it still remains underused [6, 14]. The main reason is that oral rehydration therapy neither reduces the frequency of bowel movements and fluid loss nor shortens the duration of illness, which decreases its acceptance. Effective and inexpensive interventions that could add to the effect of oral rehydration therapy are of interest to caregivers and health care professionals.

The European Society for Paediatric Gastroenterology, Hepatology and Nutrition (ESPGHAN) and the European Society of Paediatric Infectious Diseases (ESPID) recommend that select probiotics with proven clinical efficacy [e.g., *Lactobacillus* GG (LGG) or *Saccharomyces boulardii*], administered in appropriate dosages, may be used as an adjunct to rehydration therapy for the management of AGE in children [5]. The plausible mechanisms by which they may exert their effects include activation of direct inhibitors called bacteriocins, reduction of the luminal pH through short chain fatty acid production (which also inhibits some pathogens), competition for nutrients, and immunomodulatory activity [1]. Also, according to ESPGHAN/ESPID guidelines, smectite (also known as diosmectite, dioctahedral smectite), a natural hydrated aluminum magnesium silicate that binds to digestive mucous [10] and has the ability to bind endo- and exotoxins, bacteria, and rotavirus [3], may be considered in the management of AGE. In experimental models, smectite increased water and electrolyte absorption and restored the barrier properties of human intestinal cell monolayers after exposure to tumor necrosis factor-α [8]. It also modified the activity of bile salts and the physical properties of gastric mucus, thereby counteracting mucolysis induced by bacteria [10].

In countries where both LGG and smectite are available, their concomitant administration is often practiced with the intention of further reducing the length and severity of illness. However, there is uncertainty about the benefits of concomitant administration of LGG and smectite, while obviously it increases the cost of treatment. The present study was designed to determine whether LGG plus smectite is superior to LGG alone for the management of children with AGE.

## Methods

The guidelines from the CONSORT statement were followed for this study [9].

### Study design and participants

This was a double-blind, randomized, placebo-controlled trial. Children aged 4 to 60 months with AGE, defined as the passage of three or more loose or watery stools per day for >1 day but <5 days, were eligible for inclusion. Exclusion criteria included diarrhea lasting <1 or >5 days, a recent history of diarrhea indicated either by parents/guardian or hospital case notes, underlying chronic gastrointestinal disease, undernutrition (weight/height ratio below the 5th percentile), systemic infection, and immune defects or immunosuppressive treatment.

### Intervention

If a diagnosis of AGE was made, the child was assessed for eligibility and written informed consent was obtained. All patients were initially rehydrated according to the ESPGHAN and WHO recommendation (oral rehydration or, if not successful, intravenous rehydration). Regular feeding was not interrupted and was carried out after initial rehydration. At all times, breastfeeding was allowed [5, 17]. Eligible children received LGG (ATCC 53103) (commercially available as Dicoflor 30, Vitis Pharma, Poland) at a daily dosage of 6 × 10^9^ colony forming units (CFU) in one dose for 7 days plus randomly either smectite (commercially available as Smecta, Beaufour, Ipsen, France) or placebo (glucose). Both smectite and the placebo were administered as an oral dose of 3 g, once daily, until the diarrhea stopped. LGG was supplied as a powder in capsules that were opened with the contents administered in a small amount of water. The placebo and smectite were both supplied as a powder and also given in a small amount of water. While the children were in the hospital, the study products were administered by hospital staff. If a child was discharged, the study products were administered by a parent/caregiver following instructions regarding each product’s intake. In both situations, a 3-h interval between the administration of LGG and the other study products was recommended. For each child, a diary was kept to record the frequency and consistency of daily bowel movements, as well as other symptoms considered to be important or needed for analysis. The study physician assessed the patient’s diary during the follow-up visit on day 7. If a child was discharged during the study period, one of the investigators regularly contacted the family by phone to ensure adherence.

In all children, at the study entry, stool samples were collected for microbiological investigations. Standard stool cultures were used to screen for bacteria (*Salmonella*, *Shigella*, *Escherichia coli*)*.* The presence of rotavirus and enteric adenovirus types 40/41 was determined using an immunochromatographic technique (VIKIA Rota-Adeno, bioMérieux, Marcy l’Etoile, France) according to the instructions of the manufacturer.

### Outcomes

The *primary* outcome measure was the duration of diarrhea, defined as the time from randomization until the last diarrheal stool, or as at least 12 h with no stool. The *secondary* outcome measures included stool frequency, consistency of stools determined using the seven-point Bristol Stool Form Scale [7] (1 for hard lumps to 7 for watery stools—for details, see Table 1), need for antibiotic therapy (yes/no), vomiting (yes/no; how many times), diarrhea recurrence, tolerance of the study products, need for hospitalization (yes/no, how long), need for unscheduled intravenous rehydration therapy (yes/no, how long), and adverse events.

**Table 1:** Bristol Stool Form Scale [7]

Type 1	Separate hard lumps, like nuts (hard to pass)
Type 2	Sausage-shaped but lumpy
Type 3	Like a sausage but with cracks on the surface
Type 4	Like a sausage or snake, smooth and soft
Type 5	Soft blobs with clear-cut edges
Type 6	Fluffy pieces with ragged edges, a mushy stool
Type 7	Watery, no solid pieces. Entirely liquid

### Sample size

To show a difference of 24 h in duration of diarrhea with *α* = 0.05 and 80 % power (unpaired Student’s *t* test), and assuming a 20 % withdrawal rate, a total of 88 participants were needed. Sample size calculations were performed using StatsDirect (version 2.5.6; 2006-04-15 StatsDirect statistical software. http://www.statsdirect.com. England: StatsDirect Ltd. 2006).

### Randomization and blinding

Block randomization, with a block size of 8, was done with a computer-generated random number list prepared by an investigator (HS) with no clinical involvement in the trial. The list was concealed from the clinicians enrolling patients and assessing end-points (MPL, MU), as well as from the parents, until the end of the study. The study products were prepared centrally in identical packages by the hospital pharmacy at the Medical University of Warsaw by independent personnel not involved in the conduct of the trial.

### Statistical methods

The two-tailed Student’s *t* test was used for comparison of means of variables approximating a normal distribution. For non-normally distributed variables, the Mann–Whitney *U* test was used. The *χ* ^*2*^ test or Fisher exact test, as appropriate, was used for comparisons of proportions. The differences between the study groups were considered significant when the *P* value was <0.05. All analyses were conducted on an available case basis, including all participants in the groups to which they were randomized for whom outcomes were available. Statistical analyses were performed using the StatsDirect [version: 2.7.8(2011-11-09)].

## Results

### Participant flow

**Fig. 1:**
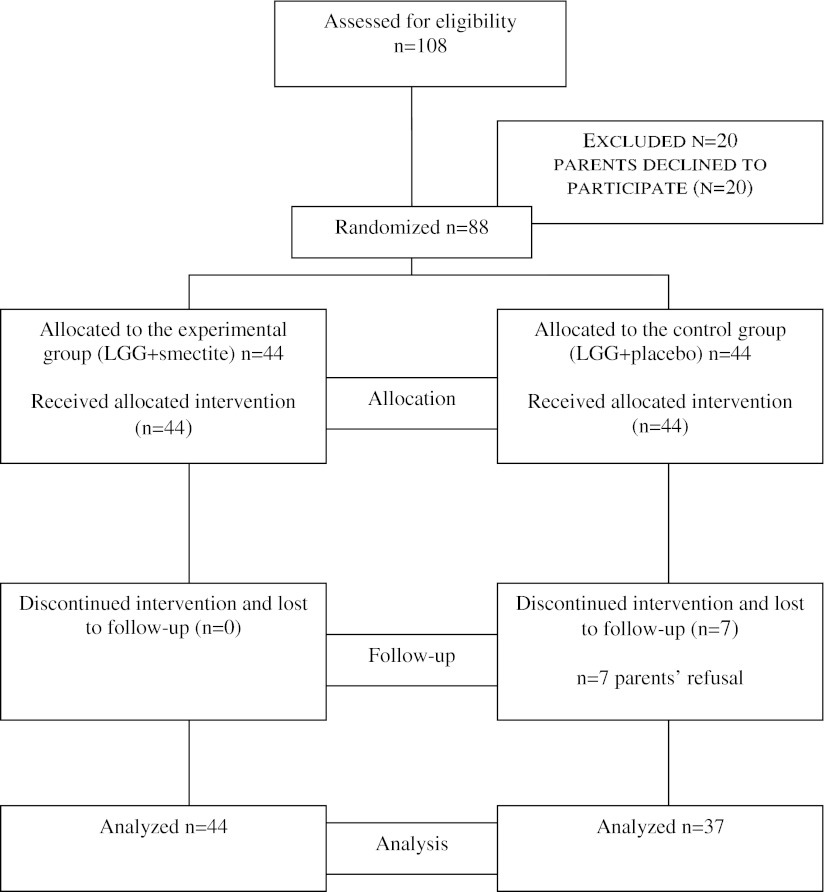
Flowchart of subjects participating in the study

Figure 1 is a flow diagram showing the subjects’ progression through the study. Of the 88 children who underwent randomization, 44 were assigned to the experimental group (LGG/smectite) and 44 were assigned to the control group (LGG/placebo). Seven children in the control group discontinued the study and eventually were lost to follow-up. A total of 81 (92 %) children were included in the analysis.

### Recruitment

Participants were recruited from among patients of the pediatric hospital of The Medical University of Warsaw, Poland, between August 2010 and June 2012.

### Baseline data

Baseline demographic and clinical characteristics did not differ between the two groups and are presented in Table 2.

**Table 2:** Study population baseline characteristics

	LGG/smectite (*n* = 44)	LGG/placebo (*n* = 44)
Male/female (*n*)	21/23	29/15
Age, months—median (range)	17.5 (10–22.5)	18 (11–30)
Weight, kg—median (range)	10 (9–12)	11 (10–14)
Inpatients/outpatients	34/10	34/10
Vomiting, *n* (%)	29 (66)	34 (77)
Vomit, episodes per day—median (range)	2.5 (0–5.25)	2 (1–4)
Dehydration, *n* (%)		
0–3 %	35 (79.5 %)	33 (75 %)
3–9 %	9 (20.5 %)	11 (25 %)
Duration of diarrhea before randomization, days—median (range)	1 (1–2.5)	2 (1–2.5)
Diarrhea etiology, *n* (%)		
Rotavirus	25 (57)	30 (68)
Adenovirus	1 (2)	3 (7)
*Salmonella*	3 (7)	1 (2)
Methicillin-sensitive *Staphylococcus aureus*	–	1 (2)
Methicillin-resistant *Staphylococcus aureus*	1 (2)	1 (2)
Enteropathogenic *E*. *coli*	1 (2)	–
*E*. *coli* extended spectrum β-lactamases	1 (2)	–
Not identified	10 (23)	6 (14)
No data available	4 (9)	5 (11)

### Outcomes

**Fig. 2:**
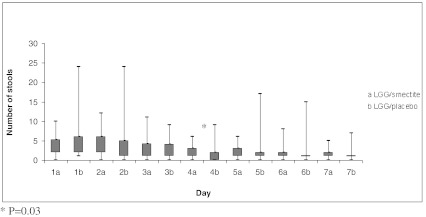
Stool frequency, number per day (median, range)

**Fig. 3:**
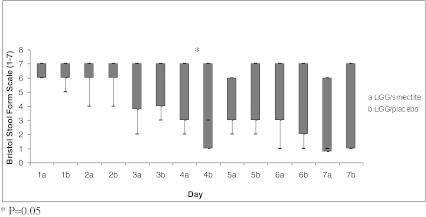
Stool consistency score using Bristol Stool Form Scale (median, range)—1 for hard lumps to 7 for watery stools

The outcome measures are summarized in Table 3 and in Figs. 2 and 3. The duration of diarrhea (the primary outcome) was similar in the LGG/smectite and LGG/placebo groups (*P* = 0.43). There were no significant differences between the study groups for the secondary outcomes, with three exceptions. On day 4, there was reduced stool frequency in the LGG/placebo group compared with the LGG/smectite group (*P* = 0.03). Also, stool consistency differed between the groups on day 4. The consistency of stools was harder in the LGG/placebo compared with the LGG/smectite group. However, stools with scores of 3, 4, and 5 on the Bristol Stool Form Scale are referred to as stools of normal consistency. No significant differences in stool consistency between groups were observed on any other day. While there was no significant difference in the need for intravenous rehydration between the groups, there was a difference in its duration. In the LGG/smectite group compared with the LGG/placebo group, there was a significantly shorter duration of intravenous therapy after randomization (*P* = 0.02). No adverse events were observed in the study groups.

**Table 3:** Primary and secondary outcome measures

	LGG/smectite (*n* = 44)	LGG/placebo (*n* = 37)	*P* value
Primary outcome			
Duration of diarrhea after randomization, days—median (range)	2 (1–3)	2 (1–2)	0.43
Secondary outcomes			
Antibiotic therapy, *n* (%)	–	–	–
Vomiting, *n* (%)	9 (20.5)	11 (30)	0.48
Vomiting, number—median (range)	0 (0–0)	0 (0–1)	0.56
Diarrhea recurrence, *n* (%)	3 (7)	3 (8)	0.83
Study product acceptance, *n* (%)	34 (77)	32 (86)	0.43
Need for hospitalization, *n* (%)	34 (77)	31 (84)	0.65
Duration of hospitalization after randomization, days—median (range)	2 (1–3)	2 (1–4)	0.19
Intravenous rehydration therapy, *n* (%)	23 (52)	25 (68)	0.24
Duration of intravenous therapy after randomization, days—median (range)	1 (0–1)	1 (0–3)	0.02

## Discussion

### Key findings

Our double-blind, randomized, placebo-controlled study showed that LGG plus smectite seems to be equally effective to LGG alone for treating children with AGE. There were no significant differences between the study groups for the secondary outcomes, with three exceptions. In the LGG/placebo group compared to the LGG/smectite group, there was significantly reduced stool frequency but only on day 4. While there was a difference between groups in stool consistency, it was not of clinical relevance; this is because stools with scores of 3, 4, and 5 on the Bristol Stool Form Scale are referred to as stools of normal consistency. If unscheduled intravenous rehydration was needed, there was a significantly shorter duration of intravenous therapy in the LGG/smectite group compared with the LGG alone group.

Several factors could explain why the addition of smectite to LGG did not provide any additional benefit over treatment with LGG alone. The first factor is the mechanism of action of smectite, including the absorption of bacteria [3]. While theoretically smectite is directed towards pathogenic bacteria, one may not exclude the possibility that it also is directed towards potentially beneficial bacteria such as LGG. However, in our trial, this explanation seems less likely, as efforts were made to administer LGG and the other study products in at least 3-h intervals. A second factor that may explain the lack of an additional benefit with smectite is that it may not have been administered at a sufficient dosage. We chose a daily dose of 3 g, as recommended by the manufacturer for children up to 1 year of age. In previous studies, the daily dose typically ranged from 3 to 6 g (depending on the age of the children) [13]; however, no clear dose–effect response has been described. Considering that the mean age of our patients included in the final analysis was 18.5 months, the dose of 3 g seems sufficient. For LGG, we chose a daily intake of 6 × 10^9^ CFU, which exceeds the minimum dose of 10^9^ CFU/day suggested in the literature for therapeutic purposes. It is noteworthy, however, that the optimal dose and treatment duration of LGG therapy have not been clearly established.

### Comparison with other studies

To our knowledge, this is the first study to assess concomitant administration of LGG with smectite. One previously published randomized controlled trial (RCT) compared administration of a mixture of three probiotic strains, i.e., *Lactobacillus rhamnosus* strains E⁄N, Oxy, and Pen (commercially available as Lakcid, Biomed, Poland), plus smectite with the administration of probiotics alone [12]. This study was carried out in 107 Polish children aged 6 to 36 months with AGE. The authors reported that in the probiotic/smectite group compared with the probiotic alone group, there was a significantly shorter duration of fluid stools, shorter duration of intravenous rehydration, shorter duration of hospitalization, shorter duration of fever >38 °C, and greater weight gain. However, there are some methodological limitations to the study, including unclear allocation concealment, no blinding, and no intention-to-treat analysis; this may result in selection, performance, and/or attrition biases and, eventually, invalidate the results.

Conversely, the efficacy of LGG and smectite administered separately has been studied in a number of RCTs and their meta-analyses. For LGG, one meta-analysis of eight RCTs, involving 988 participants, found that compared with controls, administration of LGG had no effect on the total stool volume. However, use of LGG was associated with a significant reduction in diarrhea duration, particularly that of rotaviral etiology [15]. A positive effect of using LGG for treating acute diarrhea was also confirmed in a more recent Cochrane review [1].

For smectite, one meta-analysis of data from six RCTs showed that smectite significantly reduced the duration of diarrhea compared with placebo. The chance of cure on intervention day 3 was significantly increased in the smectite versus the control group, with a number needed to treat of 4 [13]. Two subsequently performed, large, double-blind RCTs performed in Peru and Malaysia confirmed a reduction in diarrhea duration and also indicated a stool output reduction [4]. The most recent open RCT carried out in India also found that smectite reduced the duration of diarrhea and prevented a prolonged course [11].

### Strengths and limitations of the study

The strengths of this study include adequate randomization and the use of intention-to-treat analysis, both of which minimize the risk of bias. Although there was a comprehensive (92 %) follow-up, there were non-significantly more patients lost to follow-up in the LGG/placebo group compared with the LGG/smectite group (7 versus 0). As parents did not report the reasons for refusal to continue, there is no clear explanation for this finding.

Participants in this study were young children only who typically develop AGE, so the results are applicable to clinical practice. On the other hand, our results apply primarily to hospitalized children. In principle, the efficacy of the treatment may be different for various subgroups (e.g., inpatients compared with outpatients). Those admitted to the hospital may be more severely affected, later in the course of the disease, or may be more dehydrated, and thus, respond differently to treatment. Evaluation within these subgroups is warranted.

A potential limitation of our study is the lack of perfect blinding. As stated earlier, patients received smectite or placebo in small, identical packages from the hospital pharmacy. Both were white powders. However, after being dissolved in water, they were of different colors that potentially could have been an issue if the parent/caregiver had had previous experience with smectite.

To access stool consistency, we used the Bristol Stool Form Scale, which has not been validated for the youngest children. While this study was in the final planning stage, the “Amsterdam” infant stool scale was published for assessing premature and term infants’ stool consistency (four items), amount (four items), and color (six items). The scale is more detailed, and it might be helpful in differentiating between normal and abnormal defecation patterns in infants; however, it has not been validated for practical and research purposes [2]. Despite the limitations of the Bristol Stool Form Scale, it offers a more objective way of assessing stool consistency than just relying on the perceptions of caregivers.

In our trial, the duration of diarrhea was used as the primary measure of outcome. Unfortunately, this measure, alone, is not considered optimal. Quantitative diarrhea criteria are recommended by the World Health Organization for use in the evaluation of therapeutic agents for the management of acute diarrhea [16]. However, the reluctance of caregivers and health care providers to carry out the cumbersome stool collection experienced by us when conducting some previous trials was the main reason for not including stool output in this study’s protocol.

### Conclusion and implications for practice

While everyone agrees that unnecessary medicines should be avoided in the treatment of children with AGE, nevertheless some agents are commonly used. The findings from this RCT help optimize the management of AGE through the discouragement of the use of regimens with no proven efficacy but additional costs. The results of this double-blind RCT indicate that adding smectite to LGG as an adjuvant to standard rehydration therapy in infants and young children with AGE does not offer additional benefit. If there is a wish on the part of caregivers or health care professionals for an adjunct treatment, the use of either LGG or smectite is a more rational choice and may be considered.
